# QuRad: Radiomics Feature Extraction for QuPath

**DOI:** 10.34133/csbj.0238

**Published:** 2026-10-19

**Authors:** Janan Arslan, Tamara Künzle, Yeşim Güner, Agustí Alentorn

**Affiliations:** ^1^ Sorbonne Université, Institut du Cerveau—Paris Brain Institute—ICM, CNRS, Inria, Inserm, AP-HP, Hôpital de la Pitié Salpêtrière, Paris, France.; ^2^ Centre for Eye Research Australia, University of Melbourne, Royal Victorian Eye and Ear Hospital, East Melbourne, Victoria, Australia.; ^3^ LIX, École Polytechnique, Palaiseau, France.

## Abstract

Radiomic features provide a quantitative description of tissue organization in histopathology images. QuPath, a widely used open-source platform for whole-slide image analysis, offers morphology, intensity, and Haralick texture measurements but no standardized radiomic feature set such as that of PyRadiomics. Researchers must therefore export data to external Python pipelines, interrupting their QuPath workflow. QuRad extracts radiomic features from cell detections and annotation regions directly inside QuPath. The extension and equivalent Groovy script implement 119 features: 103 two-dimensional features enabled by default and 16 optional legacy shape descriptors. We compared QuRad 0.4 with PyRadiomics 3.0.1 on identical grayscale images and pixel masks. All 103 default features met a 10^−9^ absolute-or-relative agreement tolerance on a breast-cancer benchmark tile (410 cells). On 20 melanoma tiles (8,644 nuclei), 97 of 103 features met this tolerance and 6 polygon-derived shape features differed from PyRadiomics’ raster-derived mesh (minimum concordance correlation coefficient 0.942). The 22 synthetic regions accepted by PyRadiomics reproduced the expected intensity and texture values. A single-threaded server benchmark reached about 1,900 nuclei per second, and processing the 184,242 cells of a whole-slide image took 2.7 min on a laptop. A random forest distinguished tumor cells from lymphocytes in melanoma (pooled macro-F1 0.853) and classified 197 tissue regions of 6 breast-cancer slides into 3 compartments (0.696). QuRad exports the features with object coordinates and extraction settings for use in QuPath or downstream analyses.

## Introduction

Radiomics extracts quantitative features from medical images to characterize phenotypic patterns that may not be apparent through visual inspection alone. PyRadiomics [1] is a widely used open-source Python package whose feature definitions largely follow the Image Biomarker Standardization Initiative [2], with documented differences in discretization, resampling, and kurtosis. Radiomics was developed for radiology, where it has been used to study tumor heterogeneity, treatment response, sensitivity to tumor delineation, and integration with genomics [3–6]. It has also been applied to histopathology images, where texture- and intensity-based features have shown clinically relevant patterns [7,8]. In melanoma, Finti *et al*. recently classified nuclei of the PUMA (Panoptic segmentation of nUclei and tissue in advanced MelanomA) dataset with PyRadiomics and HistomicsTK features [9,10], illustrating both the interest in nucleus-level radiomics and the dependence of such analyses on external Python tooling. On the TIGER (tumor-infiltrating lymphocytes in breast cancer) breast-cancer dataset, work has focused on the computational scoring of tumor-infiltrating lymphocytes [11], including a QuPath pipeline that segments tumor and tumor-associated stroma before scoring [12].

QuPath is an open-source platform for whole-slide imaging and digital pathology [13]. It provides cell segmentation, tissue detection, object classification, and measurements of morphology, intensity, and Haralick texture, with extensions for methods such as StarDist and Cellpose [14,15]. A standardized radiomic feature set still requires exporting objects and pixels to external tools. QuRad adds this extraction directly to QuPath for cell detections and annotation regions. We compare its outputs with PyRadiomics under matched preprocessing and aggregation settings, benchmark its throughput, and demonstrate cell- and tissue-level classification. Object identifiers and coordinates accompany the measurements so they can be linked to other spatial data.

## Materials and Methods

### Experimental and technical design

The study had 4 components. We implemented radiomic features as a QuPath extension and an equivalent script; verified their numerical agreement with PyRadiomics on identical pixel masks of a benchmark tile, a second histology dataset, and synthetic images; measured the performance in a reproducible benchmark; and demonstrated their utility by the supervised classification of cells (melanoma; PUMA dataset) and tissue compartments (breast cancer; TIGER dataset) with baseline and ablation models. Figure 1B illustrates the user workflow.

**Fig. 1.**
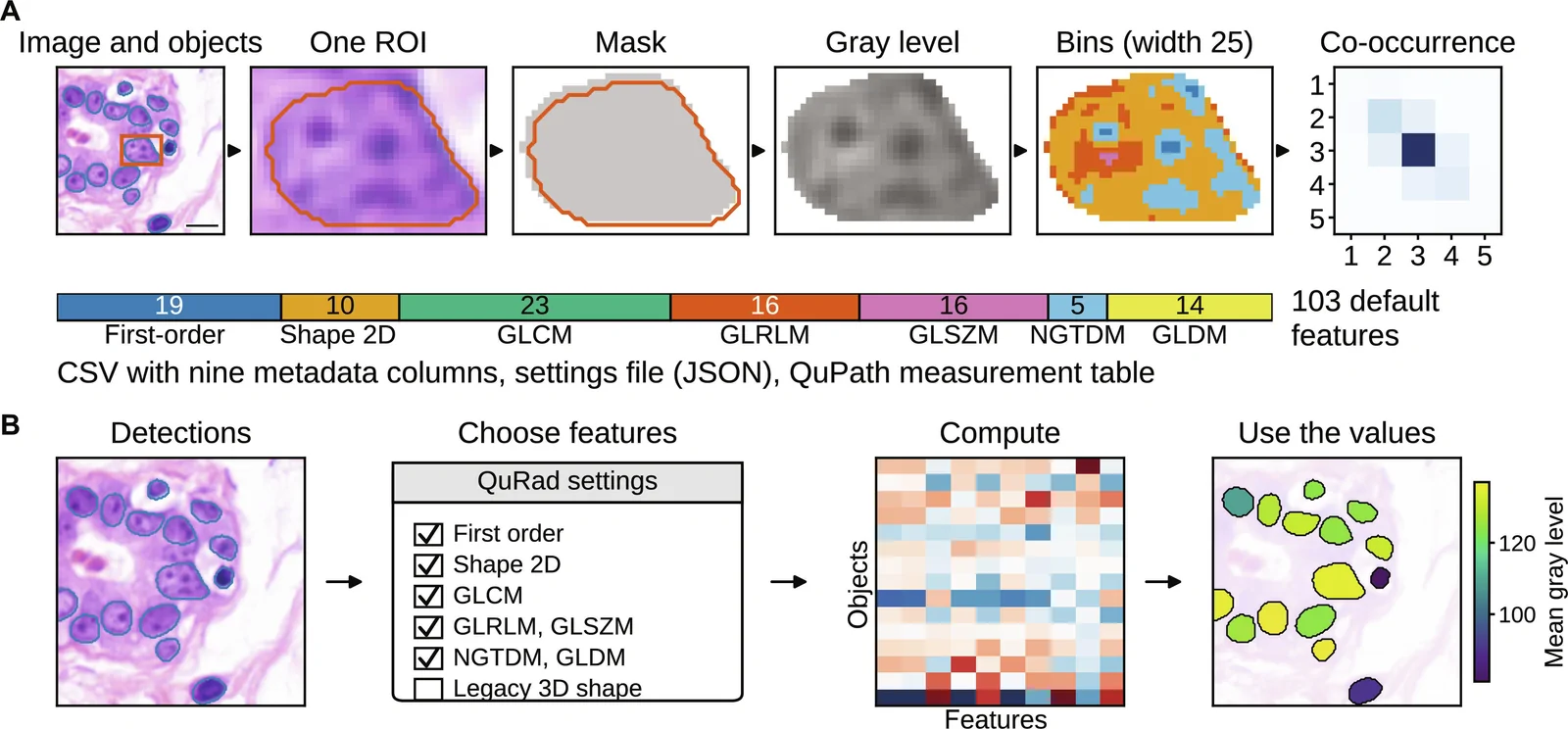
The QuRad workflow. (A) Per-object feature extraction, illustrated on one labeled cell of the breast-cancer benchmark tile: the region of the object is read at full resolution, the polygon is rasterized with the pixel-center rule (orange outline), the pixels are converted to gray levels and discretized with a fixed bin width, and the texture matrices are accumulated over the 4 in-plane directions. The co-occurrence matrix of this object is shown with discretized gray-level bins on both axes, and the bar gives the composition of the 103 features computed by default. Scale bar, 20 μm. (B) The same run as the user performs it in QuPath with the default settings: the image holds the cell detections (blue), the feature classes are chosen in the dialog, every detection is measured, and the values are used inside QuPath, here to shade each nucleus by its mean gray level.

### Software implementation

QuRad is distributed as an installable QuPath extension and as a self-contained Groovy script that share the same feature-extraction code (version 0.4.0 here). QuRad builds only on QuPath’s Java API and the Java standard library and needs no external plugins or libraries. The extension adds a menu command with a settings dialog and works with QuPath 0.6 and 0.7. The script is opened and run in QuPath’s script editor. Figure 1A gives an overview of the extraction workflow.

#### Feature set

QuRad implements 119 features in 8 classes (Table 1). The 103 features enabled by default are 2-dimensional quantities, and each has a direct PyRadiomics equivalent: first-order statistics (19), 2-dimensional shape (10), gray-level co-occurrence matrix (GLCM, 23), gray-level run-length matrix (GLRLM, 16), gray-level size-zone matrix (GLSZM, 16), neighborhood gray-tone difference matrix (NGTDM, 5), and gray-level dependence matrix (GLDM, 14). Feature names follow the pattern class_FeatureName. Prefixing original_ gives the PyRadiomics name, so exported columns can be mapped programmatically. The eighth class (shape, 16 features) is optional and disabled by default. It reports 2-dimensional quantities under PyRadiomics’ 3-dimensional shape names, for example, shape_MeshVolume for the polygon area, so that pipelines expecting those column names keep working. PyRadiomics computes its 3-dimensional shape features on a single slice as a one-voxel-thick volume, so its values differ from QuRad’s 2-dimensional quantities. The dialog and the documentation therefore mark these features as legacy. Table S1 gives the feature mapping and validation results, and the online Feature Reference defines each feature.

**Table 1.** Parameters and conventions of QuRad 0.4 and the PyRadiomics 3.0.1 configuration that reproduces them

Aspect	QuRad	PyRadiomics setting
Input	8-bit RGB brightfield image, read at full resolution (other pixel types refused)	force2D=True
Grayscale	$\left\lfloor \frac{\left(299R+587G+114B\right)}{1,000}\right\rfloor$, integer arithmetic	Same conversion applied before extraction
Pixel mask	Pixel included if its center lies inside the ROI polygon (Java2D fill of the exact geometry, no stroke widening or anti-aliasing), clipped to the image	Label mask
Discretization	Fixed bin width 25 (configurable), edges at multiples of the width from 0	binWidth=25, normalize=False
Intensity shift	0	voxelArrayShift=0
GLCM	Symmetric, distance 1 px (configurable), 4 directions (0°, 45°, 90°, and 135°), matrices summed	symmetricalGLCM=True, distances=[1], weightingNorm='no_weighting'
GLRLM	Same 4 directions, matrices summed	weightingNorm='no_weighting'
GLSZM, GLDM, NGTDM	8-connectivity, distance 1, α=0	Defaults
Percentiles, median	Linear interpolation	Default
Undefined values	Flat region: entropy 0, uniformity 1, skewness and kurtosis 0, GLCM correlation 1, informational measures of correlation (IMC1 and IMC2) 0, coarseness 10^6^, and 0 for any other zero denominator	Same
Default classes	First-order 19, shape2D 10, GLCM 23, GLRLM 16, GLSZM 16, NGTDM 5, GLDM 14 (103 features)	All enabled explicitly, including optional features
Optional class	Legacy 3D-named shape (16), off by default	Not comparable (one-voxel-thick volume)
Metadata	Image, object ID, type, class, centroid, pixel count, pixel calibration, settings JSON file	–
Threads	1	–

#### Conventions

Table 1 lists every parameter and convention needed to reproduce QuRad’s output. All of them are also written to a machine-readable settings file next to each comma-separated values (CSV) file. QuRad accepts 8-bit RGB brightfield images and refuses other pixel types. Pixels are read at full resolution and converted to a gray level $g=\left\lfloor \left(299R+587G+114B\right)/1,000\right\rfloor$ with integer arithmetic. A pixel belongs to a region of interest (ROI) if its center lies inside the ROI polygon. QuRad implements this rule with a Java2D fill of the exact polygon geometry, without stroke widening or anti-aliasing, and pixels outside the image bounds are ignored. Intensities are discretized with a fixed bin width (default 25), with bin edges at multiples of the bin width from zero, as in PyRadiomics. GLCMs are symmetric and are accumulated over the 4 in-plane directions (0°, 45°, 90°, and 135°) at a configurable pixel distance (default 1). GLRLMs are accumulated over the same 4 directions. In both cases, the matrices are summed before the features are computed. This corresponds to the PyRadiomics setting weightingNorm='no_weighting', whereas the PyRadiomics default averages the feature values over the directions. Size zones and dependences use 8-connectivity with α=0. Undefined quantities, such as those of a flat region, follow PyRadiomics (Table 1). Objects whose polygon contains no pixel center are skipped, and the number skipped is reported. Distances and lengths are in pixels, and the pixel calibration is recorded in the output so that texture scale can be related to physical units.

#### Output

QuRad writes one CSV per image. It contains the features and 9 metadata columns: image name, object identifier, object type, classification, centroid coordinates, number of pixels used, and pixel width and height in micrometers. A JSON file next to it records the software and QuPath versions and all settings. Optionally, the features are also inserted into QuPath’s measurement list, for measurement maps and interactive exploration. The feature routines are statically compiled Groovy code that operates on dense integer matrices. Extraction is single-threaded and deterministic.

### Datasets

#### Numerical validation

We used 3 kinds of input. (a) A single breast-cancer tissue-microarray tile (896 × 768 pixels, 410 labeled cells) from the UCSB Bio-Segmentation benchmark [16], whose label image provides a raster ground truth for the masks. (b) The first 20 primary-melanoma tiles of the PUMA training set [17] (ROIs 001 to 020, 1,024×1,024 pixels, hematoxylin and eosin [H&E], 8,649 annotated nuclei). They come from a different scanner and a different tissue, and their polygons are hand-annotated and vary in size and shape. (c) Twenty-five synthetic ROIs on deterministic images (uniform, checkerboard, gradient, 2-level, noise and random-color images). They comprise square, circular, elongated, star-shaped, ring-shaped, sub-pixel-offset, border-touching and border-crossing polygons, ROIs of 1 to 25 pixels, and ROIs of up to 512 × 512 pixels. Table S2 lists them, and for several of them, the feature values are known analytically.

#### Classification

From PUMA, we used the same 20 tiles and kept tumor cells (n=4,130) and lymphocytes (n=2,056), 6,186 nuclei in total. The other cell types were not used. From the TIGER breast-cancer dataset [11,18], we used the region annotations of 6 whole-slide images (100B, 154B, 198B, 207B, 234B, and 242B). We kept invasive tumor (n=103), tumor-associated stroma (n=62), and healthy glands (n=32), 197 regions in total. Classes with fewer than 30 annotations (in situ tumor, necrosis, and a residual “rest” category) were excluded. Healthy glands occur in only 2 of the 6 slides (100B and 198B). The classification feature tables of both datasets were produced with QuRad 0.4 as an extension in QuPath 0.7, with the nucleus (PUMA) and region (TIGER) annotations imported as QuPath objects. The PUMA objects are annotated nuclei imported into QuPath, whereas the whole-slide timing experiment uses automatically detected cells. The PUMA tables agree with the command-line runner output used for the numerical validation to within 3 × 10^−15^ on every nucleus.

#### Human and animal research

This study used only publicly available, de-identified image datasets and involved no new experimentation on humans or animals. The validation, PUMA, and TIGER datasets were obtained from their respective public repositories (see Data Availability).

### Numerical validation against PyRadiomics

Both implementations were given identical inputs, so that only the feature computation is compared. A command-line runner distributed with the source code uses the same calculator as the extension and the script on an image and a set of GeoJSON objects. Its output agrees with that of the extension run inside QuPath (see the “Datasets” section). For every object, it exports the pixel mask it used and the grayscale image it computed. PyRadiomics 3.0.1 was then run on these masks and this grayscale image with the settings of Table 1, with all optional features enabled (firstorder_StandardDeviation, shape2D_SphericalDisproportion, shape_Compactness1/2, and shape_SphericalDisproportion) and with weightingNorm='no_weighting'. A second PyRadiomics run used its default angle aggregation, to measure the effect of this setting. For the benchmark tile, the GeoJSON polygons were traced from the label image with marching squares and shifted by half a pixel into QuPath’s coordinate system. The pixel-center rule must then reproduce the label image exactly. We verified this for every cell, and we verified that the grayscale images computed in Java and in NumPy are identical.

For every feature we computed, across objects, Pearson’s *r*, Lin’s concordance correlation coefficient (CCC) [19], the intraclass correlation coefficient ICC(2,1) for absolute agreement [20], the mean, median, and maximum absolute error, the median and maximum relative error, the slope and intercept of QuRad regressed on PyRadiomics, and Bland–Altman statistics [21] (Table S1 and Figs. S1 and S2). A feature meets the agreement tolerance when its maximum absolute or maximum relative error over all objects is below 10^−9^, with relative errors computed only where the PyRadiomics value exceeds 10^−12^ in magnitude. This threshold is larger than double-precision rounding errors (about 10^−16^) and smaller than the differences caused by a change of convention, such as the angle aggregation (at least 2 × 10^−3^, see the “Numerical agreement with PyRadiomics” section). Unit tests in the source tree additionally check analytically derived values for uniform, checkerboard, and small synthetic inputs.

### Performance benchmark

We timed QuRad on a complete whole-slide image and in a scaling experiment. On TIGER slide 100B (67,456 × 37,376 pixels), the cells were detected with QuPath’s built-in cell detection (default 5 μm cell expansion) and processed by the extension in QuPath 0.7 on a laptop (Apple M2, 16 GB). In the scaling experiment, the command-line runner processed the 634 nuclei of PUMA tile 001 in 1 to 40 passes (634 to 25,360 objects, nucleus areas 23 to 1,568 pixels, median 275) on an AMD EPYC 7643 server (OpenJDK 21.0.11). QuRad runs on one thread. Times cover region reading, rasterization, and the computation of the 103 default features. CSV writing was timed separately, and insertion into the measurement list is not included. Memory is the sum of the peak use of the JVM heap pools. The Supplementary Materials give the full protocol and 2 further experiments, on ROI size and on single feature classes (Fig. S3 and Table S3).

### Classification analysis

Classification was performed with scikit-learn 1.7.2 [22] using a Pipeline whose data-dependent steps are fitted on the training fold only and then applied to the held-out fold: median imputation, removal of constant features, greedy pruning of feature pairs with $\left|r\right|>0.90$ (the feature with the higher mean absolute correlation to all others is dropped), and standardization. The classifier was a random forest [23] with 500 trees, class_weight='balanced', a minimum split size of 2 and a maximum depth of 15 (PUMA) or 10 (TIGER). The hyperparameters were fixed a priori and not tuned, and the random seeds were 42 (PUMA) and 0 (TIGER). Cross-validation was grouped by image, so that no image contributed to both the training and the test fold. PUMA used 5 folds of 4 tiles. The 6 TIGER slides were split into 3 folds, which GroupKFold filled with 1, 2, and 3 slides to balance the number of regions, and were also evaluated leave-one-slide-out. Fold assignments are listed in Table S4. Both main models used the 103 default features. Ablations repeated the pipeline on 7 feature sets: (a) 10 basic morphology and intensity measurements comparable to QuPath’s built-in cell measurements (area, perimeter, major and minor axis, elongation, sphericity, mean, standard deviation, and minimum and maximum intensity), taken from the QuRad output so that they are computed on the same pixel masks as the other sets; (b) first-order features only; (c) 2-dimensional shape only; (d) texture only; (e) first-order plus shape; (f) first-order plus texture without any shape feature; and (g) all features including the legacy shape class. Feature importance was summarized as the mean decrease in impurity averaged over folds, together with the number of folds in which a feature survived the correlation filter (Table S5). Figure S4 shows the correlation structure of the unfiltered features, ordered by class. Low-dimensional structure was visualized with Uniform Manifold Approximation and Projection (UMAP) [24] on the standardized, correlation-pruned features of a class-balanced subsample of 3,000 PUMA nuclei and of the 197 TIGER regions. Class signatures were shown with a clustered heatmap of the mean *z*-scored features per class [25].

### Statistical analysis

Agreement statistics are described above and are reported per feature in Table S1. Over features, we report the median and minimum of *r*, CCC, and ICC; the number of features within tolerance; and the largest relative error. Classification performance is the macro-F1 of the pooled out-of-fold predictions over all task classes. The mean ± SD of the fold macro-F1 describes the variation between held-out sets, and Table S4 explains how folds that lack a class are scored. Out-of-fold confusion matrices are shown in Fig. S5, per-fold results are shown in Table S4, and per-image results are shown in Table S6.

## Results

### Numerical agreement with PyRadiomics

On the benchmark tile, the pixel masks used by QuRad were identical to the label image for all 410 cells, and the grayscale images computed in Java and in NumPy were identical pixel for pixel. Under matched settings, all 103 default features met the combined 10^−9^ tolerance. The largest relative error was about 1.0 × 10^−9^, for NGTDM contrast, whose absolute error was below the 10^−9^ threshold. The median of the per-feature maxima was 6 × 10^−16^. Pearson’s *r*, CCC, and ICC(2,1) were therefore 1.000 for every feature, and the regression slopes and intercepts were 1 and 0 to numerical precision (Table 2, Table S1, and Figs. S1 and S2).

**Table 2.** Feature-wise agreement between QuRad 0.4 and PyRadiomics 3.0.1 on identical masks (default 103 features, matched angle aggregation). Within tolerance: maximum absolute error or maximum relative error <10^−9^, with reference magnitudes ≤10^−12^ excluded from relative errors. PUMA excludes 5 nuclei refused by PyRadiomics. ICC(2,1) equals the CCC to 3 decimals in all cases. The 6 polygon-based shape features are shape2D_MeshSurface, shape2D_Perimeter, shape2D_PerimeterSurfaceRatio, shape2D_Sphericity, shape2D_SphericalDisproportion, and shape2D_MaximumDiameter.

Dataset	Objects	Features within tolerance	Median (min) *r*	Median (min) CCC	Largest relative error
UCSB breast tile	410	103/103	1.000 (1.000)	1.000 (1.000)	1.0×10^−9^ (NGTDM contrast)
PUMA, 20 tiles	8,644	97/103	1.000 (0.955)	1.000 (0.942)	0.38 (maximum diameter)
Synthetic ROIs	22	97–98/103	–	–	Polygon-based shape features only

On the PUMA tiles, PyRadiomics refused 5 of the 8,649 extracted nuclei because they were narrower than 2 pixels, so 8,644 nuclei were compared. Of the 103 features, 97 met the tolerance. The 6 remaining ones are the polygon-based shape features. QuRad measures the annotation polygon as drawn, whereas PyRadiomics reconstructs a marching-squares mesh from the raster, which cuts the corners of a pixelated outline. For polygons traced from the label image with marching squares, as on the benchmark tile, both coincide exactly. For the hand-drawn PUMA polygons, the perimeter differed by a median of 1.6% in either direction (CCC 0.996), the area differed by 0.3% (CCC 1.000), and the sphericity differed by 1.7% (CCC 0.94). The synthetic shapes show the same effect (Table S2): for a 20 × 20 square, the mesh gives an area of 399.5 and a perimeter of 78.8 against 400 and 80 for the polygon, and for a circle of radius 15, the mesh perimeter is 6% longer. PyRadiomics accepted 22 of the 25 synthetic ROIs and refused the 1-pixel, 1 × 2-pixel and 1 × 40-pixel ones. On all 22, every intensity and texture feature met the tolerance, including the uniform, near-uniform, one-bin, border-crossing, ring-shaped, and 512 × 512-pixel regions. All analytically expected values were reproduced: for example, entropy 0 and joint energy 1 for a uniform region, mean 50 and entropy 1 for a 0/100 checkerboard, and coarseness 10^6^ for a flat region.

#### Angle aggregation

We also compared QuRad with the PyRadiomics default, which averages the feature values over the 4 directions. Under that convention, 65 of the 103 features met the tolerance on the benchmark tile, but 38 GLCM and GLRLM features differed. The median relative error was 0.4% for the GLCM features and 2.6% for the GLRLM features, and the differences were much larger for individual cells (up to 46% for glrlm_LongRunEmphasis). The nonnormalized nonuniformity features glrlm_GrayLevelNonUniformity and glrlm_RunLengthNonUniformity were approximately 4-fold larger on this tile when the matrices were summed over 4 directions. Users who compare QuRad with PyRadiomics defaults must therefore expect these differences.

Of the 16 legacy 3D-named shape features, 6 coincide numerically with PyRadiomics on the benchmark tile: shape_VoxelVolume, shape_MajorAxisLength, shape_MinorAxisLength, shape_Elongation, shape_Maximum3DDiameter, and shape_Maximum2DDiameterSlice. On the hand-drawn PUMA polygons, the last 2 also differ. The other 10 do not coincide. For example, shape_Sphericity has r=−0.08, shape_SurfaceArea has CCC 0.02, and shape_LeastAxisLength is constant 0 in PyRadiomics. This is expected because PyRadiomics evaluates the mask as a one-voxel-thick volume. Table S1 lists them.

### Throughput

On the complete TIGER slide, QuRad processed 184,242 cells (mean 559 pixels) in 161.6 s, or 1,140 cells per second on the laptop, including reading the tiles from the slide file. In the server scaling experiment, the throughput rose from 283 objects per second for 634 nuclei, with just-in-time compilation overhead, to 1,900 objects per second for 25,360 nuclei (Fig. S3). The largest run used 910 MiB of heap memory, and writing its CSV took 1.32 s.

### Radiomic features distinguish tumor cells from lymphocytes

With fold-internal preprocessing, 62 to 63 of the 103 default features survived the correlation filter in each fold. The random forest separated tumor cells from lymphocytes with a pooled out-of-fold macro-F1 of 0.853 (mean over folds 0.838 ± 0.022, Table 3 and Fig. 2A). The per-tile macro-F1 ranged from 0.62 to 0.95. Across all PUMA nuclei, the most internally redundant classes are shape2D (20% of within-class pairs with $\left|r\right|>0.9$), first-order (12%), and GLCM (10%). GLRLM, GLSZM, NGTDM, and GLDM contain few redundant pairs (at most 2.2%, Fig. S4). The most important features were firstorder_Energy, shape2D_MajorAxisLength, glszm_LargeAreaHighGrayLevelEmphasis, glrlm_GrayLevelNonUniformity, and gldm_DependenceVariance, each retained in all 5 folds (Table S5). Lymphocytes have smaller and darker nuclei, hence lower energy, a shorter major axis and fewer large bright zones, and a more heterogeneous dependence structure. In the ablation (Table 4), texture features alone reach a macro-F1 of 0.839; first-order plus shape features, 0.855; and the 10 basic measurements, 0.843; thus, these feature sets performed similarly in this task. Including the legacy shape class changed the pooled score by 0.001. The UMAP of the unsupervised, correlation-pruned feature space shows only a gradual separation of the 2 populations (Fig. 2B).

**Table 3.** Classification performance with fold-internal preprocessing. Precision, recall, and F1 are computed on the pooled out-of-fold predictions, and fold macro-F1 is given as mean ± SD over folds. PUMA: 5-fold grouped by tile, n=6,186 nuclei. TIGER: 3-fold grouped by slide, n=197 regions.

Dataset	Class	Precision	Recall	F1
PUMA	Lymphocyte	0.82	0.78	0.80
PUMA	Tumor	0.89	0.92	0.90
PUMA	Macro average	0.86	0.85	0.853
PUMA	Fold macro-F1	–	–	0.838 ± 0.022
TIGER	Healthy glands	0.75	0.66	0.70
TIGER	Invasive tumor	0.70	0.81	0.75
TIGER	Tumor-associated stroma	0.71	0.58	0.64
TIGER	Macro average	0.72	0.68	0.696
TIGER	Fold macro-F1	–	–	0.694 ± 0.057

**Fig. 2.**
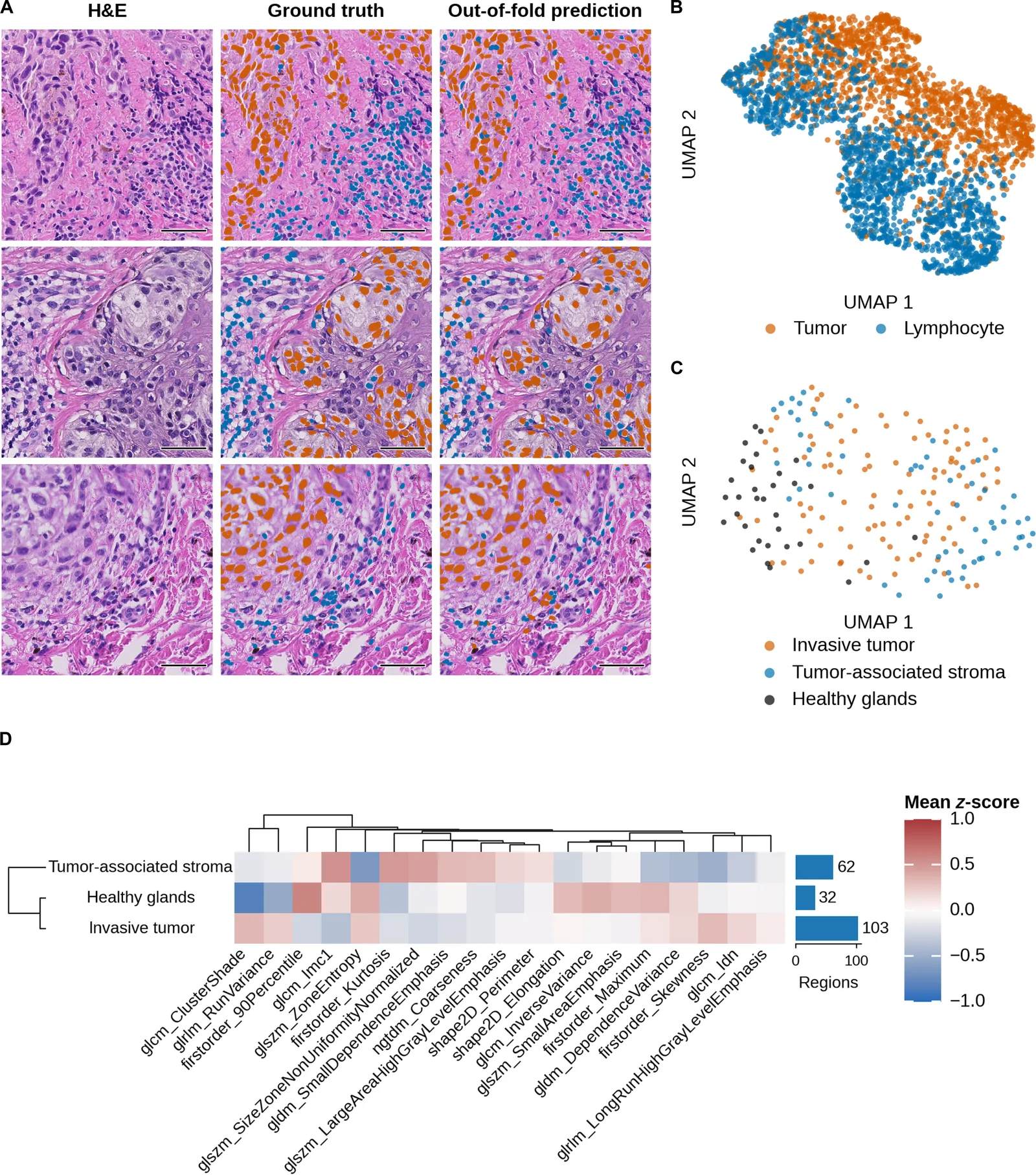
Application of QuRad features. (A) Three PUMA melanoma tiles (001, 004, and 006, 1,024×1,024 pixels at 0.227 μm per pixel) showing the H&E image, the ground-truth annotations, and the out-of-fold random-forest predictions (tumor = vermilion, lymphocyte = blue, pooled macro-F1 = 0.85 over all 20 tiles). Scale bars, 50 μm. (B) Unsupervised UMAP of the standardized, correlation-pruned QuRad features of a class-balanced subsample of 3,000 PUMA nuclei. (C) Unsupervised UMAP of the 197 TIGER regions (invasive tumor = vermilion, tumor-associated stroma = blue, healthy glands = black). (D) Mean *z*-scored value per tissue type of the 19 features retained by the correlation filter in every fold, with hierarchical clustering of rows and columns. The bars give the number of regions per class.

**Table 4.** Ablation over feature families (pooled out-of-fold macro-F1, with the mean ± SD over folds in brackets). “Basic” denotes 10 morphology and intensity measurements comparable to QuPath’s built-in cell measurements, computed on the same masks.

Feature set	Features	PUMA	TIGER
Basic morphology/intensity	10	0.843 [0.83 ± 0.02]	0.651 [0.66 ± 0.03]
First-order only	19	0.832 [0.82 ± 0.02]	0.676 [0.68 ± 0.04]
Shape 2D only	10	0.766 [0.75 ± 0.02]	0.538 [0.53 ± 0.15]
Texture only	74	0.839 [0.83 ± 0.03]	0.711 [0.71 ± 0.08]
First-order + shape 2D	29	0.855 [0.84 ± 0.02]	0.663 [0.66 ± 0.03]
First-order + texture	93	0.838 [0.82 ± 0.02]	0.694 [0.69 ± 0.03]
All nonlegacy features	103	0.853 [0.84 ± 0.02]	0.696 [0.69 ± 0.06]
All + legacy shape	119	0.854 [0.84 ± 0.02]	0.701 [0.69 ± 0.08]

### Radiomic features classify tissue compartments

On the TIGER regions, 37 to 43 features survived the fold-internal filter. The random forest reached a pooled macro-F1 of 0.696 under 3-fold cross-validation grouped by slide (mean over folds 0.694 ± 0.057, Table 3). Leave-one-slide-out evaluation gave a pooled macro-F1 of 0.671 with a wide spread over folds (0.60 ± 0.16). Healthy glands are present in only 2 slides, so when one of them is held out, the model must recognize glands from the other slide alone. In the ablation (Table 4), shape features alone reach only 0.54. Texture features alone reach 0.711; intensity plus texture without any shape feature, 0.694; and the full model, 0.696. The baseline of 10 basic measurements reaches 0.651. Apart from shape alone, the differences between these feature sets are within the spread over folds. The leading model feature was glcm_DifferenceVariance, retained in 2 of 3 folds, while firstorder_90Percentile was retained in all 3 (Table S5). The UMAP of individual regions shows partial separation, because the regions within each compartment vary widely (Fig. 2C). The class means, shown as *z*-scores in the clustered heatmap, nevertheless form a distinct signature for each compartment (Fig. 2D). In the class medians over all regions, tumor-associated stroma is the brightest and most homogeneous compartment: the median of the mean gray level is 156 versus 134 in invasive tumor; the 10th percentile, 108 versus 77; and the interquartile range, 41 versus 61. This fits eosinophilic collagen with sparse nuclei. Healthy glands combine the strongest local contrast and cluster prominence (GLCM contrast 1.02 versus 0.66 in tumor, cluster prominence 453 versus 247) with a wide intensity spread, which aligns with the alternation of dark, densely packed epithelial nuclei with bright lumina and stroma. Invasive tumor regions are the darkest, with intermediate texture, as expected from high nuclear density and the loss of glandular architecture.

## Discussion

Under matched settings, all 103 default features met the numerical tolerance on the breast-cancer benchmark, as did all intensity and texture features on the PUMA nuclei and on the synthetic regions that PyRadiomics accepted. Six shape features differed on hand-drawn polygons because QuRad measures the original polygon, whereas PyRadiomics reconstructs a mesh from its pixel mask. GLCM and GLRLM feature values also depend on how the 4 directions are combined, so QuRad records its matrix-summation convention in the exported settings.

For the melanoma nuclei, the radiomic features separated tumor cells from lymphocytes, but the 10 basic measurements performed about as well. For the breast-cancer tissue regions, texture alone scored highest, removing the shape features changed little, and the class profiles aligned with the known histology of each compartment. These are point estimates without significance tests, and several intensity and texture features also depend on ROI size. Finti *et al*. [9] reported higher performance for PyRadiomics than for HistomicsTK in their PUMA tumor-versus-nontumor experiment, but their labels and evaluation differ from ours, so the scores are not directly comparable.

The classification experiments are limited to a deterministic subset of 20 PUMA tiles and 6 TIGER slides, with healthy glands present in only 2 slides. Grouping by PUMA tile does not guarantee independence between patients, and there was no external validation set. The implementation accepts luminance-converted 8-bit RGB images only, without stain-separated channels, higher bit depths, or PyRadiomics’ filtered inputs. All features use native-resolution pixels. Adjusting the GLCM distance changes only its sampling offset and does not harmonize the other texture families or pixel-based shape measurements across resolutions, so such comparisons require a consistent spatial scale. Future work could add stain-specific channels and parallel extraction, and HistomicsTK’s nucleus-level and stain-specific measurements [10] could complement the current feature set.

In summary, QuRad brings a standardized radiomic feature set into the QuPath workflow, and its default feature values closely match PyRadiomics on identical pixel masks under matched settings. Its features distinguished tumor cells from lymphocytes and classified 3 tissue compartments of breast cancer, in line with earlier radiomic studies of histology [7,9]. They can carry biologically meaningful information, which users can explore directly in QuPath.

## Data Availability

The QuRad source code and documentation are openly available at https://github.com/institutducerveau/QuRad under the MIT license. The version evaluated here, 0.4.0, is archived on Zenodo (https://doi.org/10.5281/zenodo.23062835). The repository contains the feature tables of all 3 datasets, the PyRadiomics comparison tables, the benchmark timings, the 3 notebooks and the helper scripts that produce every table and figure panel of the results, and their outputs. The image used for validation is a single breast-cancer tissue-microarray tile from the UCSB Bio-Segmentation benchmark [16], available under a Creative Commons Attribution 3.0 (CC BY 3.0) license and redistributed with attribution. The biological application datasets are publicly available: the PUMA melanoma dataset [17] from the PUMA Grand Challenge (https://puma.grand-challenge.org/) and the TIGER breast-cancer dataset [18] from the TIGER Grand Challenge (https://tiger.grand-challenge.org/).
