# Supplementary material for: QuRad: Radiomics Feature Extraction for QuPath
Source: Comput Struct Biotechnol J. 2026 Oct 19;35(1):0238. doi: 10.34133/csbj.0238 (PMC13678076; doi:10.34133/csbj.0238)
Supplement: Supplementary file 1 [file csbj.0238.f1.pdf]

# Supplementary Material

QuRad: Radiomics Feature Extraction for QuPath

Janan Arslan, Tamara Künzle, Yeşim Güner, Agustí Alentorn

## Contents


- Table S1: mapping of all QuRad features to PyRadiomics and per-feature agreement on identical masks.
- Table S2: synthetic validation cases.
- Table S3: benchmark results.
- Table S4: fold assignments, class counts and results of each fold.
- Table S5: feature importance and selection frequency.
- Table S6: per-image classification results.
- Figure S1: per-feature maximum relative error on both validation datasets.
- Figure S2: scatter and Bland–Altman plots for nine representative features.
- Figure S3: benchmark.
- Figure S4: feature–feature correlation matrices.
- Figure S5: out-of-fold confusion matrices.

# Table S1: feature mapping and agreement

The columns give the QuRad feature name, whether the feature is enabled by default, Pearson's $r$, the concordance correlation coefficient (CCC) and the maximum relative error over the 410 cells of the UCSB benchmark tile, the CCC and the maximum relative error over the 8 644 compared PUMA nuclei (8 649 extracted, five refused by PyRadiomics), and the agreement status on both datasets. The PyRadiomics name is `original_` followed by the QuRad name. A feature passes when its maximum absolute error or its maximum relative error is below $10^{-9}$, with reference magnitudes $\leq 10^{-12}$ excluded from relative errors, and "diff." marks a feature outside this tolerance. Maximum relative errors below $10^{-12}$ are shown as 0. Legacy 3D-named shape features are 2D quantities and are not expected to agree with PyRadiomics' evaluation of the mask as a one-voxel-thick volume.

| QuRad feature | Default | UCSB $r$ | UCSB CCC | UCSB max err | PUMA CCC | PUMA max err | status UCSB / PUMA |
|---|---|---|---|---|---|---|---|
| firstorder_10Percentile | yes | 1.0000 | 1.0000 | 0 | 1.0000 | 0 | pass / pass |
| firstorder_90Percentile | yes | 1.0000 | 1.0000 | 0 | 1.0000 | 0 | pass / pass |
| firstorder_Energy | yes | 1.0000 | 1.0000 | 0 | 1.0000 | 0 | pass / pass |
| firstorder_Entropy | yes | 1.0000 | 1.0000 | 0 | 1.0000 | 0 | pass / pass |
| firstorder_InterquartileRange | yes | 1.0000 | 1.0000 | 0 | 1.0000 | 0 | pass / pass |
| firstorder_Kurtosis | yes | 1.0000 | 1.0000 | 0 | 1.0000 | 0 | pass / pass |
| firstorder_Maximum | yes | 1.0000 | 1.0000 | 0 | 1.0000 | 0 | pass / pass |
| firstorder_Mean | yes | 1.0000 | 1.0000 | 0 | 1.0000 | 0 | pass / pass |
| firstorder_MeanAbsoluteDeviation | yes | 1.0000 | 1.0000 | 0 | 1.0000 | 0 | pass / pass |
| firstorder_Median | yes | 1.0000 | 1.0000 | 0 | 1.0000 | 0 | pass / pass |
| firstorder_Minimum | yes | 1.0000 | 1.0000 | 0 | 1.0000 | 0 | pass / pass |
| firstorder_Range | yes | 1.0000 | 1.0000 | 0 | 1.0000 | 0 | pass / pass |
| firstorder_RobustMeanAbsoluteDeviation | yes | 1.0000 | 1.0000 | 0 | 1.0000 | 0 | pass / pass |
| firstorder_RootMeanSquared | yes | 1.0000 | 1.0000 | 0 | 1.0000 | 0 | pass / pass |
| firstorder_Skewness | yes | 1.0000 | 1.0000 | 0 | 1.0000 | $2.6 \times 10^{-12}$ | pass / pass |
| firstorder_StandardDeviation | yes | 1.0000 | 1.0000 | 0 | 1.0000 | 0 | pass / pass |
| firstorder_TotalEnergy | yes | 1.0000 | 1.0000 | 0 | 1.0000 | 0 | pass / pass |
| firstorder_Uniformity | yes | 1.0000 | 1.0000 | 0 | 1.0000 | 0 | pass / pass |
| firstorder_Variance | yes | 1.0000 | 1.0000 | 0 | 1.0000 | 0 | pass / pass |
| glcm_Autocorrelation | yes | 1.0000 | 1.0000 | 0 | 1.0000 | 0 | pass / pass |
| glcm_ClusterProminence | yes | 1.0000 | 1.0000 | 0 | 1.0000 | 0 | pass / pass |
| glcm_ClusterShade | yes | 1.0000 | 1.0000 | $3.0 \times 10^{-12}$ | 1.0000 | $2.3 \times 10^{-11}$ | pass / pass |
| glcm_ClusterTendency | yes | 1.0000 | 1.0000 | 0 | 1.0000 | 0 | pass / pass |
| glcm_Contrast | yes | 1.0000 | 1.0000 | 0 | 1.0000 | 0 | pass / pass |
| glcm_Correlation | yes | 1.0000 | 1.0000 | 0 | 1.0000 | 0 | pass / pass |
| glcm_DifferenceAverage | yes | 1.0000 | 1.0000 | 0 | 1.0000 | 0 | pass / pass |
| glcm_DifferenceEntropy | yes | 1.0000 | 1.0000 | 0 | 1.0000 | 0 | pass / pass |
| glcm_DifferenceVariance | yes | 1.0000 | 1.0000 | 0 | 1.0000 | 0 | pass / pass |
| glcm_Id | yes | 1.0000 | 1.0000 | 0 | 1.0000 | 0 | pass / pass |
| glcm_Idm | yes | 1.0000 | 1.0000 | 0 | 1.0000 | 0 | pass / pass |
| glcm_Idmn | yes | 1.0000 | 1.0000 | 0 | 1.0000 | 0 | pass / pass |
| glcm_Idn | yes | 1.0000 | 1.0000 | 0 | 1.0000 | 0 | pass / pass |
| glcm_Imc1 | yes | 1.0000 | 1.0000 | 0 | 1.0000 | $1.7 \times 10^{-12}$ | pass / pass |
| glcm_Imc2 | yes | 1.0000 | 1.0000 | $1.1 \times 10^{-12}$ | 1.0000 | 0 | pass / pass |
| glcm_InverseVariance | yes | 1.0000 | 1.0000 | 0 | 1.0000 | 0 | pass / pass |
| glcm_JointAverage | yes | 1.0000 | 1.0000 | 0 | 1.0000 | 0 | pass / pass |
| glcm_JointEnergy | yes | 1.0000 | 1.0000 | 0 | 1.0000 | 0 | pass / pass |
| glcm_JointEntropy | yes | 1.0000 | 1.0000 | 0 | 1.0000 | 0 | pass / pass |
| glcm_MaximumProbability | yes | 1.0000 | 1.0000 | 0 | 1.0000 | 0 | pass / pass |
| glcm_SumAverage | yes | 1.0000 | 1.0000 | 0 | 1.0000 | 0 | pass / pass |
| glcm_SumEntropy | yes | 1.0000 | 1.0000 | 0 | 1.0000 | 0 | pass / pass |

| QuRad feature | Default | UCSB $r$ | UCSB CCC | UCSB max err | PUMA CCC | PUMA max err | status UCSB / PUMA |
|---|---|---|---|---|---|---|---|
| glcm_SumSquares | yes | 1.0000 | 1.0000 | 0 | 1.0000 | 0 | pass / pass |
| gldm_DependenceEntropy | yes | 1.0000 | 1.0000 | 0 | 1.0000 | 0 | pass / pass |
| gldm_DependenceNonUniformity | yes | 1.0000 | 1.0000 | 0 | 1.0000 | 0 | pass / pass |
| gldm_DependenceNonUniformityNormalized | yes | 1.0000 | 1.0000 | 0 | 1.0000 | 0 | pass / pass |
| gldm_DependenceVariance | yes | 1.0000 | 1.0000 | 0 | 1.0000 | 0 | pass / pass |
| gldm_GrayLevelNonUniformity | yes | 1.0000 | 1.0000 | 0 | 1.0000 | 0 | pass / pass |
| gldm_GrayLevelVariance | yes | 1.0000 | 1.0000 | 0 | 1.0000 | 0 | pass / pass |
| gldm_HighGrayLevelEmphasis | yes | 1.0000 | 1.0000 | 0 | 1.0000 | 0 | pass / pass |
| gldm_LargeDependenceEmphasis | yes | 1.0000 | 1.0000 | 0 | 1.0000 | 0 | pass / pass |
| gldm_LargeDependenceHighGrayLevelEmphasis | yes | 1.0000 | 1.0000 | 0 | 1.0000 | 0 | pass / pass |
| gldm_LargeDependenceLowGrayLevelEmphasis | yes | 1.0000 | 1.0000 | 0 | 1.0000 | 0 | pass / pass |
| gldm_LowGrayLevelEmphasis | yes | 1.0000 | 1.0000 | 0 | 1.0000 | 0 | pass / pass |
| gldm_SmallDependenceEmphasis | yes | 1.0000 | 1.0000 | 0 | 1.0000 | 0 | pass / pass |
| gldm_SmallDependenceHighGrayLevelEmphasis | yes | 1.0000 | 1.0000 | 0 | 1.0000 | 0 | pass / pass |
| gldm_SmallDependenceLowGrayLevelEmphasis | yes | 1.0000 | 1.0000 | 0 | 1.0000 | 0 | pass / pass |
| glrlm_GrayLevelNonUniformity | yes | 1.0000 | 1.0000 | 0 | 1.0000 | 0 | pass / pass |
| glrlm_GrayLevelNonUniformityNormalized | yes | 1.0000 | 1.0000 | 0 | 1.0000 | 0 | pass / pass |
| glrlm_GrayLevelVariance | yes | 1.0000 | 1.0000 | 0 | 1.0000 | 0 | pass / pass |
| glrlm_HighGrayLevelRunEmphasis | yes | 1.0000 | 1.0000 | 0 | 1.0000 | 0 | pass / pass |
| glrlm_LongRunEmphasis | yes | 1.0000 | 1.0000 | 0 | 1.0000 | 0 | pass / pass |
| glrlm_LongRunHighGrayLevelEmphasis | yes | 1.0000 | 1.0000 | 0 | 1.0000 | 0 | pass / pass |
| glrlm_LongRunLowGrayLevelEmphasis | yes | 1.0000 | 1.0000 | 0 | 1.0000 | 0 | pass / pass |
| glrlm_LowGrayLevelRunEmphasis | yes | 1.0000 | 1.0000 | 0 | 1.0000 | 0 | pass / pass |
| glrlm_RunEntropy | yes | 1.0000 | 1.0000 | 0 | 1.0000 | 0 | pass / pass |
| glrlm_RunLengthNonUniformity | yes | 1.0000 | 1.0000 | 0 | 1.0000 | 0 | pass / pass |
| glrlm_RunLengthNonUniformityNormalized | yes | 1.0000 | 1.0000 | 0 | 1.0000 | 0 | pass / pass |
| glrlm_RunPercentage | yes | 1.0000 | 1.0000 | 0 | 1.0000 | 0 | pass / pass |
| glrlm_RunVariance | yes | 1.0000 | 1.0000 | 0 | 1.0000 | 0 | pass / pass |
| glrlm_ShortRunEmphasis | yes | 1.0000 | 1.0000 | 0 | 1.0000 | 0 | pass / pass |
| glrlm_ShortRunHighGrayLevelEmphasis | yes | 1.0000 | 1.0000 | 0 | 1.0000 | 0 | pass / pass |
| glrlm_ShortRunLowGrayLevelEmphasis | yes | 1.0000 | 1.0000 | 0 | 1.0000 | 0 | pass / pass |
| glszm_GrayLevelNonUniformity | yes | 1.0000 | 1.0000 | 0 | 1.0000 | 0 | pass / pass |
| glszm_GrayLevelNonUniformityNormalized | yes | 1.0000 | 1.0000 | 0 | 1.0000 | 0 | pass / pass |
| glszm_GrayLevelVariance | yes | 1.0000 | 1.0000 | 0 | 1.0000 | 0 | pass / pass |
| glszm_HighGrayLevelZoneEmphasis | yes | 1.0000 | 1.0000 | 0 | 1.0000 | 0 | pass / pass |
| glszm_LargeAreaEmphasis | yes | 1.0000 | 1.0000 | 0 | 1.0000 | 0 | pass / pass |
| glszm_LargeAreaHighGrayLevelEmphasis | yes | 1.0000 | 1.0000 | 0 | 1.0000 | 0 | pass / pass |
| glszm_LargeAreaLowGrayLevelEmphasis | yes | 1.0000 | 1.0000 | 0 | 1.0000 | 0 | pass / pass |
| glszm_LowGrayLevelZoneEmphasis | yes | 1.0000 | 1.0000 | 0 | 1.0000 | 0 | pass / pass |
| glszm_SizeZoneNonUniformity | yes | 1.0000 | 1.0000 | 0 | 1.0000 | 0 | pass / pass |
| glszm_SizeZoneNonUniformityNormalized | yes | 1.0000 | 1.0000 | 0 | 1.0000 | 0 | pass / pass |
| glszm_SmallAreaEmphasis | yes | 1.0000 | 1.0000 | 0 | 1.0000 | 0 | pass / pass |
| glszm_SmallAreaHighGrayLevelEmphasis | yes | 1.0000 | 1.0000 | 0 | 1.0000 | 0 | pass / pass |
| glszm_SmallAreaLowGrayLevelEmphasis | yes | 1.0000 | 1.0000 | 0 | 1.0000 | 0 | pass / pass |
| glszm_ZoneEntropy | yes | 1.0000 | 1.0000 | 0 | 1.0000 | 0 | pass / pass |
| glszm_ZonePercentage | yes | 1.0000 | 1.0000 | 0 | 1.0000 | 0 | pass / pass |
| glszm_ZoneVariance | yes | 1.0000 | 1.0000 | 0 | 1.0000 | 0 | pass / pass |
| ngtdm_Busyness | yes | 1.0000 | 1.0000 | 0 | 1.0000 | 0 | pass / pass |
| ngtdm_Coarseness | yes | 1.0000 | 1.0000 | 0 | 1.0000 | 0 | pass / pass |
| ngtdm_Complexity | yes | 1.0000 | 1.0000 | 0 | 1.0000 | 0 | pass / pass |
| ngtdm_Contrast | yes | 1.0000 | 1.0000 | $1.0 \times 10^{-9}$ | 1.0000 | $2.4 \times 10^{-9}$ | pass / pass |
| ngtdm_Strength | yes | 1.0000 | 1.0000 | 0 | 1.0000 | 0 | pass / pass |
| shape2D_Elongation | yes | 1.0000 | 1.0000 | 0 | 1.0000 | 0 | pass / pass |

| QuRad feature | Default | UCSB $r$ | UCSB CCC | UCSB max err | PUMA CCC | PUMA max err | status UCSB / PUMA |
|---|---|---|---|---|---|---|---|
| shape2D_MajorAxisLength | yes | 1.0000 | 1.0000 | 0 | 1.0000 | 0 | pass / pass |
| shape2D_MaximumDiameter | yes | 1.0000 | 1.0000 | 0 | 0.9996 | 0.375 | pass / diff. |
| shape2D_MeshSurface | yes | 1.0000 | 1.0000 | 0 | 1.0000 | 0.158 | pass / diff. |
| shape2D_MinorAxisLength | yes | 1.0000 | 1.0000 | 0 | 1.0000 | 0 | pass / pass |
| shape2D_Perimeter | yes | 1.0000 | 1.0000 | 0 | 0.9956 | 0.327 | pass / diff. |
| shape2D_PerimeterSurfaceRatio | yes | 1.0000 | 1.0000 | 0 | 0.9954 | 0.282 | pass / diff. |
| shape2D_PixelSurface | yes | 1.0000 | 1.0000 | 0 | 1.0000 | 0 | pass / pass |
| shape2D_SphericalDisproportion | yes | 1.0000 | 1.0000 | 0 | 0.9498 | 0.280 | pass / diff. |
| shape2D_Sphericity | yes | 1.0000 | 1.0000 | 0 | 0.9418 | 0.219 | pass / diff. |
| shape_Compactness1 | no | -0.7101 | -0.0022 | 46.700 | -0.0025 | 204.437 | diff. / diff. |
| shape_Compactness2 | no | -0.6847 | $-3.4 \times 10^{-4}$ | 2274.245 | $-2.4 \times 10^{-4}$ | 42203.287 | diff. / diff. |
| shape_Elongation | no | 1.0000 | 1.0000 | 0 | 1.0000 | 0 | pass / pass |
| shape_LeastAxisLength | no | n/a | 0 | n/a | 0 | n/a | diff. / diff. |
| shape_MajorAxisLength | no | 1.0000 | 1.0000 | 0 | 1.0000 | 0 | pass / pass |
| shape_Maximum2DDiameterColumn | no | 0.9032 | 0.8694 | 1.375 | 0.9340 | 1.556 | diff. / diff. |
| shape_Maximum2DDiameterRow | no | 0.9042 | 0.8703 | 1.625 | 0.9331 | 1.611 | diff. / diff. |
| shape_Maximum2DDiameterSlice | no | 1.0000 | 1.0000 | 0 | 0.9996 | 0.375 | pass / diff. |
| shape_Maximum3DDiameter | no | 1.0000 | 1.0000 | 0 | 0.9996 | 0.375 | pass / diff. |
| shape_MeshVolume | no | 0.9999 | 0.9901 | 0.251 | 0.9978 | 0.941 | diff. / diff. |
| shape_MinorAxisLength | no | 1.0000 | 1.0000 | 0 | 1.0000 | 0 | pass / pass |
| shape_SphericalDisproportion | no | -0.0913 | -0.0023 | 0.699 | -0.0014 | 0.845 | diff. / diff. |
| shape_Sphericity | no | -0.0773 | -0.0018 | 2.322 | -0.0019 | 5.435 | diff. / diff. |
| shape_SurfaceArea | no | 0.9284 | 0.0222 | 0.922 | 0.0240 | 0.971 | diff. / diff. |
| shape_SurfaceVolumeRatio | no | 0.9978 | 0.0041 | 0.925 | 0.0040 | 0.971 | diff. / diff. |
| shape_VoxelVolume | no | 1.0000 | 1.0000 | 0 | 1.0000 | 0 | pass / pass |

## Table S2: synthetic validation cases

For every synthetic ROI: number of pixels, number of default features compared with PyRadiomics, number of features within tolerance, and the features outside tolerance (all of which are the boundary-dependent 2D shape features, for which QuRad measures the polygon and PyRadiomics a marching-squares mesh of the raster). ROIs refused by PyRadiomics (fewer than two pixels in one dimension) are listed with the refusal reason. The validation notebook checks the analytically expected values of these cases.

| Image | ROI | Pixels | Compared | Pass | Difference / note |
|---|---|---|---|---|---|
| border | border_topleft | 400 | 103 | 97 | 6 polygon shape |
| border | border_right | 400 | 103 | 97 | 6 polygon shape |
| border | border_crossing | 256 | 103 | 97 | 6 polygon shape |
| checkerboard | checker_square | 400 | 103 | 97 | 6 polygon shape |
| colour | colour_circle | 1264 | 103 | 97 | 6 polygon shape |
| colour | colour_square | 900 | 103 | 97 | 6 polygon shape |
| gradient | gradient_rect | 1920 | 103 | 97 | 6 polygon shape |
| large | large_512 | 262144 | 103 | 97 | 6 polygon shape |
| large | large_circle | 196350 | 103 | 97 | 6 polygon shape |
| nearuniform | near_uniform_same_bin | 225 | 103 | 97 | 6 polygon shape |
| nearuniform | near_uniform_two_bins | 900 | 103 | 97 | 6 polygon shape |
| noise | noise_circle | 712 | 103 | 97 | 6 polygon shape |
| noise | noise_bar_1x40 | 40 | 0 | 0 | PyRadiomics refusal |
| noise | noise_bar_2x40 | 80 | 103 | 97 | 6 polygon shape |
| noise | noise_diagonal | 160 | 103 | 98 | 5 polygon shape |
| noise | noise_star | 493 | 103 | 97 | 6 polygon shape |
| noise | noise_ring | 800 | 103 | 98 | 5 polygon shape |
| noise | noise_subpixel_square | 315 | 103 | 97 | 6 polygon shape |
| tiny | tiny_1px | 1 | 0 | 0 | PyRadiomics refusal |
| tiny | tiny_1x2 | 2 | 0 | 0 | PyRadiomics refusal |
| tiny | tiny_2x2 | 4 | 103 | 97 | 6 polygon shape |
| tiny | tiny_3x3 | 9 | 103 | 97 | 6 polygon shape |
| tiny | tiny_5x5 | 25 | 103 | 97 | 6 polygon shape |
| twolevel | two_level | 1600 | 103 | 97 | 6 polygon shape |
| uniform | uniform_square | 900 | 103 | 97 | 6 polygon shape |

## Table S3: benchmark

Single-threaded extraction of the 103 default features with the command-line runner on an AMD EPYC 7643 virtual server (OpenJDK 21.0.11, QuPath 0.6.0 libraries). A run labeled $\times n$ processes the objects of a tile in $n$ passes. Times are measured after a three-object warm-up and exclude CSV writing, which is timed separately, and insertion into the measurement list. Heap use is the sum of the peak use of the JVM heap pools in MiB, excluding native memory.

| Run | Objects | Extraction (s) | Objects/s | CSV (s) | Heap peaks (MiB) |
|---|---|---|---|---|---|
| PUMA tile 001 × 1 | 634 | 2.24 | 283 | 0.13 | 211 |
| PUMA tile 001 × 2 | 1268 | 2.93 | 433 | 0.19 | 221 |
| PUMA tile 001 × 5 | 3170 | 4.45 | 713 | 0.29 | 274 |
| PUMA tile 001 × 10 | 6340 | 6.96 | 910 | 0.51 | 494 |
| PUMA tile 001 × 10 (+ legacy shape) | 6340 | 6.50 | 975 | 0.50 | 556 |
| PUMA tile 001 × 20 | 12680 | 9.33 | 1358 | 0.75 | 705 |
| PUMA tile 001 × 40 | 25360 | 13.34 | 1900 | 1.32 | 910 |
| UCSB tile × 5 | 2050 | 3.27 | 628 | 0.25 | 195 |
| Single feature class enabled (PUMA tile 001 × 5): ms per nucleus | | | | | |
| firstorder 0.53; glcm 0.79; gldm 0.49; glrlm 0.72; glszm 0.67; ngtdm 0.48; shape 0.50; shape2D 0.52 | | | | | |
| Square ROIs on noise (median of 10): side, ms per ROI | | | | | |
| 8: 3.19; 16: 3.66; 32: 3.92; 64: 5.07; 128: 11.95; 256: 31.27; 512: 105.53; 1024: 407.47 | | | | | |

## Table S4: classification folds

Fold assignment, sample sizes, retained features, number of evaluated classes and the macro-F1 of each fold. “TIGER LOWO” denotes the leave-one-slide-out evaluation. Fold macro-F1 averages the classes that occur in the held-out labels or predictions (the scikit-learn default), so a class that is predicted but absent from the labels counts with F1 zero. Healthy glands are absent from TIGER fold 2 and from leave-one-slide-out folds 2, 4, 5 and 6, so these folds do not test gland recognition.

| Dataset | Fold | Held-out tiles/slides | Train | Test | Kept | Classes | Macro-F1 |
|---|---|---|---|---|---|---|---|
| PUMA | 1 | 3, 6, 10, 13 | 4945 | 1241 | 63 | 2 | 0.856 |
| PUMA | 2 | 12, 16, 17, 20 | 4962 | 1224 | 62 | 2 | 0.805 |
| PUMA | 3 | 4, 5, 9, 11 | 4976 | 1210 | 63 | 2 | 0.840 |
| PUMA | 4 | 1, 7, 8, 18 | 4940 | 1246 | 62 | 2 | 0.861 |
| PUMA | 5 | 2, 14, 15, 19 | 4921 | 1265 | 63 | 2 | 0.829 |
| TIGER | 1 | 198B | 128 | 69 | 37 | 3 | 0.687 |
| TIGER | 2 | 207B, 234B | 132 | 65 | 39 | 2 | 0.641 |
| TIGER | 3 | 100B, 154B, 242B | 134 | 63 | 43 | 3 | 0.755 |
| TIGER LOWO | 1 | 100B | 175 | 22 | 45 | 3 | 0.476 |
| TIGER LOWO | 2 | 154B | 178 | 19 | 45 | 2 | 0.474 |
| TIGER LOWO | 3 | 198B | 128 | 69 | 37 | 3 | 0.687 |
| TIGER LOWO | 4 | 207B | 138 | 59 | 41 | 2 | 0.642 |
| TIGER LOWO | 5 | 234B | 191 | 6 | 45 | 2 | 0.486 |
| TIGER LOWO | 6 | 242B | 175 | 22 | 43 | 2 | 0.861 |

## Table S5: feature importance and selection frequency

The fifteen leading features per task, ranked by mean decrease in impurity averaged over all training folds. Importance is set to zero in folds where a feature is not retained. The final column gives the number of folds retaining the feature (out of five for PUMA and three for TIGER). Importances are not class-specific.

| Dataset | Feature | Mean importance | Folds retained |
|---|---|---|---|
| PUMA | firstorder_Energy | 0.1049 | 5 |
| PUMA | shape2D_MajorAxisLength | 0.0679 | 5 |
| PUMA | glszm_LargeAreaHighGrayLevelEmphasis | 0.0623 | 5 |
| PUMA | glrlm_GrayLevelNonUniformity | 0.0535 | 5 |
| PUMA | gldm_DependenceVariance | 0.0390 | 5 |
| PUMA | firstorder_Skewness | 0.0375 | 5 |
| PUMA | ngtdm_Strength | 0.0372 | 5 |
| PUMA | glcm_ClusterShade | 0.0366 | 5 |
| PUMA | ngtdm_Coarseness | 0.0346 | 5 |
| PUMA | glrlm_LongRunHighGrayLevelEmphasis | 0.0256 | 5 |
| PUMA | firstorder_RootMeanSquared | 0.0253 | 4 |
| PUMA | glrlm_RunLengthNonUniformityNormalized | 0.0239 | 4 |
| PUMA | shape2D_PerimeterSurfaceRatio | 0.0219 | 5 |
| PUMA | gldm_DependenceNonUniformity | 0.0215 | 4 |
| PUMA | gldm_LargeDependenceHighGrayLevelEmphasis | 0.0213 | 5 |
| TIGER | glcm_DifferenceVariance | 0.0463 | 2 |
| TIGER | firstorder_90Percentile | 0.0347 | 3 |
| TIGER | glcm_DifferenceEntropy | 0.0332 | 2 |
| TIGER | firstorder_10Percentile | 0.0328 | 2 |
| TIGER | glcm_SumEntropy | 0.0317 | 2 |
| TIGER | firstorder_Kurtosis | 0.0315 | 3 |
| TIGER | glszm_SmallAreaEmphasis | 0.0310 | 3 |
| TIGER | glcm_ClusterProminence | 0.0300 | 2 |
| TIGER | gldm_SmallDependenceEmphasis | 0.0285 | 3 |
| TIGER | glcm_Imc1 | 0.0284 | 3 |
| TIGER | glcm_ClusterShade | 0.0283 | 3 |
| TIGER | glszm_LargeAreaLowGrayLevelEmphasis | 0.0280 | 2 |
| TIGER | firstorder_InterquartileRange | 0.0275 | 2 |
| TIGER | glcm_Contrast | 0.0269 | 1 |
| TIGER | firstorder_Skewness | 0.0241 | 3 |

## Table S6: per-image classification results

Out-of-fold performance for each PUMA tile and TIGER slide. "TIGER" uses the three-fold model and "TIGER LOWO" the leave-one-slide-out predictions. Per-image macro-F1 averages all task classes, and a class absent from both the labels and the predictions of a slide counts as zero, so a TIGER slide without glands scores at most 0.67. This differs from the convention for folds in Table S4.

| Dataset | Tile/slide | Objects | Classes present | Accuracy | Macro-F1 |
|---|---|---|---|---|---|
| PUMA | 1 | 405 | 2 | 0.859 | 0.855 |
| PUMA | 2 | 410 | 2 | 0.980 | 0.745 |
| PUMA | 3 | 617 | 2 | 0.880 | 0.866 |
| PUMA | 4 | 215 | 2 | 0.809 | 0.804 |
| PUMA | 5 | 324 | 2 | 0.873 | 0.751 |
| PUMA | 6 | 156 | 2 | 0.878 | 0.875 |
| PUMA | 7 | 163 | 2 | 0.706 | 0.615 |
| PUMA | 8 | 475 | 2 | 0.943 | 0.710 |
| PUMA | 9 | 553 | 2 | 0.826 | 0.812 |
| PUMA | 10 | 243 | 2 | 0.798 | 0.755 |
| PUMA | 11 | 118 | 2 | 0.873 | 0.871 |
| PUMA | 12 | 133 | 2 | 0.962 | 0.954 |
| PUMA | 13 | 225 | 2 | 0.853 | 0.756 |
| PUMA | 14 | 183 | 2 | 0.945 | 0.900 |
| PUMA | 15 | 445 | 2 | 0.906 | 0.842 |
| PUMA | 16 | 573 | 2 | 0.808 | 0.669 |
| PUMA | 17 | 198 | 2 | 0.869 | 0.856 |
| PUMA | 18 | 203 | 2 | 0.921 | 0.917 |
| PUMA | 19 | 227 | 2 | 0.815 | 0.745 |
| PUMA | 20 | 320 | 2 | 0.887 | 0.864 |
| TIGER | 100B | 22 | 3 | 0.727 | 0.641 |
| TIGER | 154B | 19 | 2 | 0.579 | 0.342 |
| TIGER | 198B | 69 | 3 | 0.696 | 0.687 |
| TIGER | 207B | 59 | 2 | 0.712 | 0.428 |
| TIGER | 234B | 6 | 2 | 0.667 | 0.417 |
| TIGER | 242B | 22 | 2 | 0.864 | 0.574 |
| TIGER LOWO | 100B | 22 | 3 | 0.591 | 0.476 |
| TIGER LOWO | 154B | 19 | 2 | 0.526 | 0.316 |
| TIGER LOWO | 198B | 69 | 3 | 0.696 | 0.687 |
| TIGER LOWO | 207B | 59 | 2 | 0.712 | 0.428 |
| TIGER LOWO | 234B | 6 | 2 | 0.500 | 0.324 |
| TIGER LOWO | 242B | 22 | 2 | 0.864 | 0.574 |

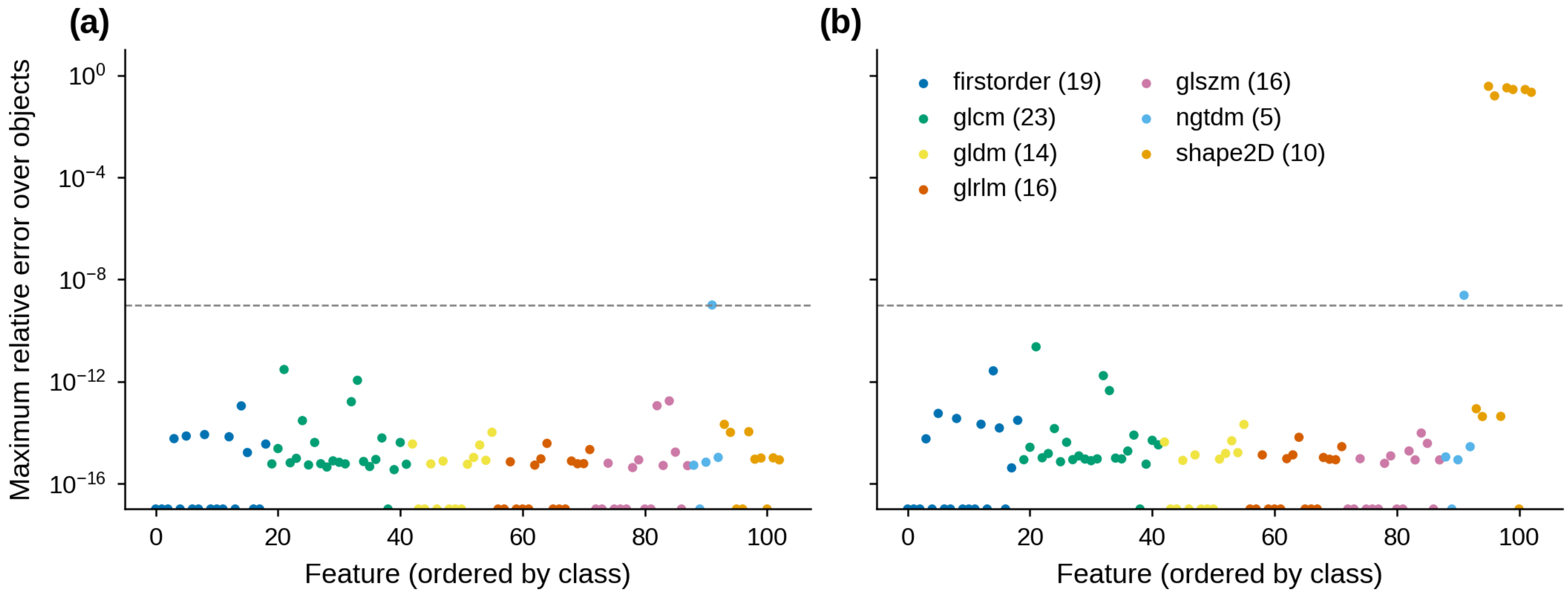


Figure S1: Maximum relative error over objects for every default feature, on (a) the UCSB benchmark tile and (b) the PUMA tiles, colored by feature class. The dashed line marks the relative-error criterion of the $10^{-9}$ agreement tolerance, and the alternative absolute-error criterion is defined in Methods. The six polygon-based shape features exceed the tolerance on PUMA. NGTDM contrast also lies above the dashed line but passes the absolute criterion (maximum absolute error $5.34 \times 10^{-11}$ on UCSB and $9.12 \times 10^{-11}$ on PUMA).

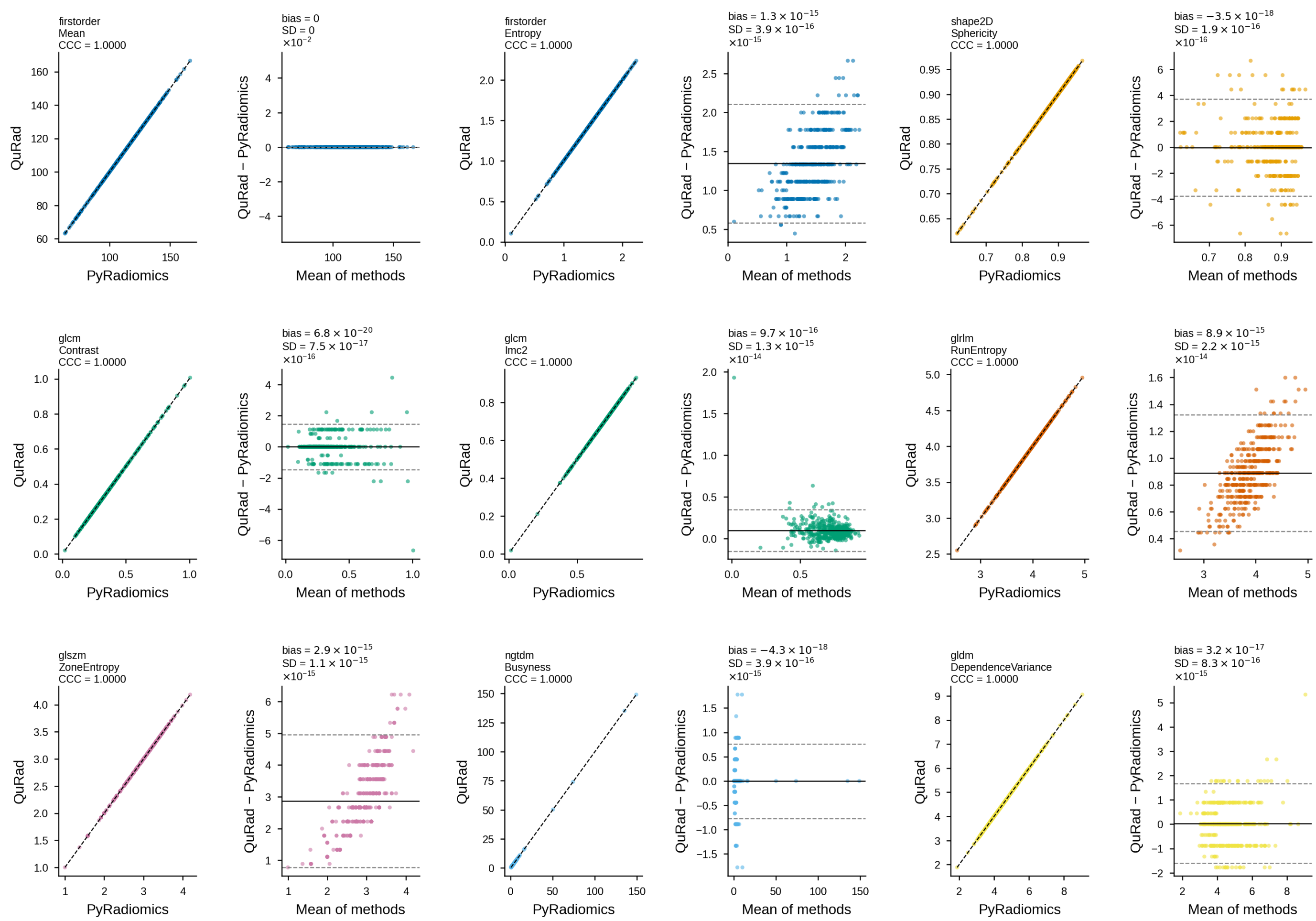


Figure S2: Scatter plots against the identity line (left of each pair) and Bland–Altman plots (right of each pair) for nine representative features on the UCSB benchmark tile.

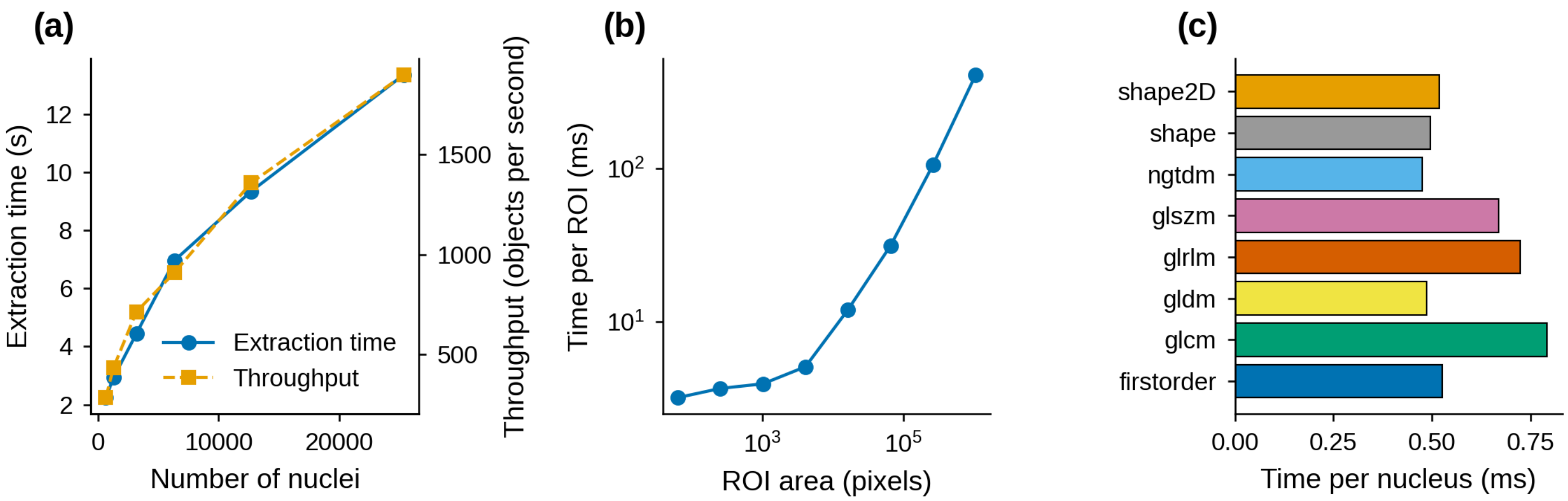


Figure S3: Benchmark: (a) extraction time and throughput versus number of nuclei (the 634 nuclei of PUMA tile 001 processed in 1–40 passes), (b) time per ROI versus ROI area (square ROIs on a noise image, median of ten repetitions), and (c) time per nucleus with a single feature class enabled (five passes over PUMA tile 001, including the fixed cost of reading and rasterizing each region).

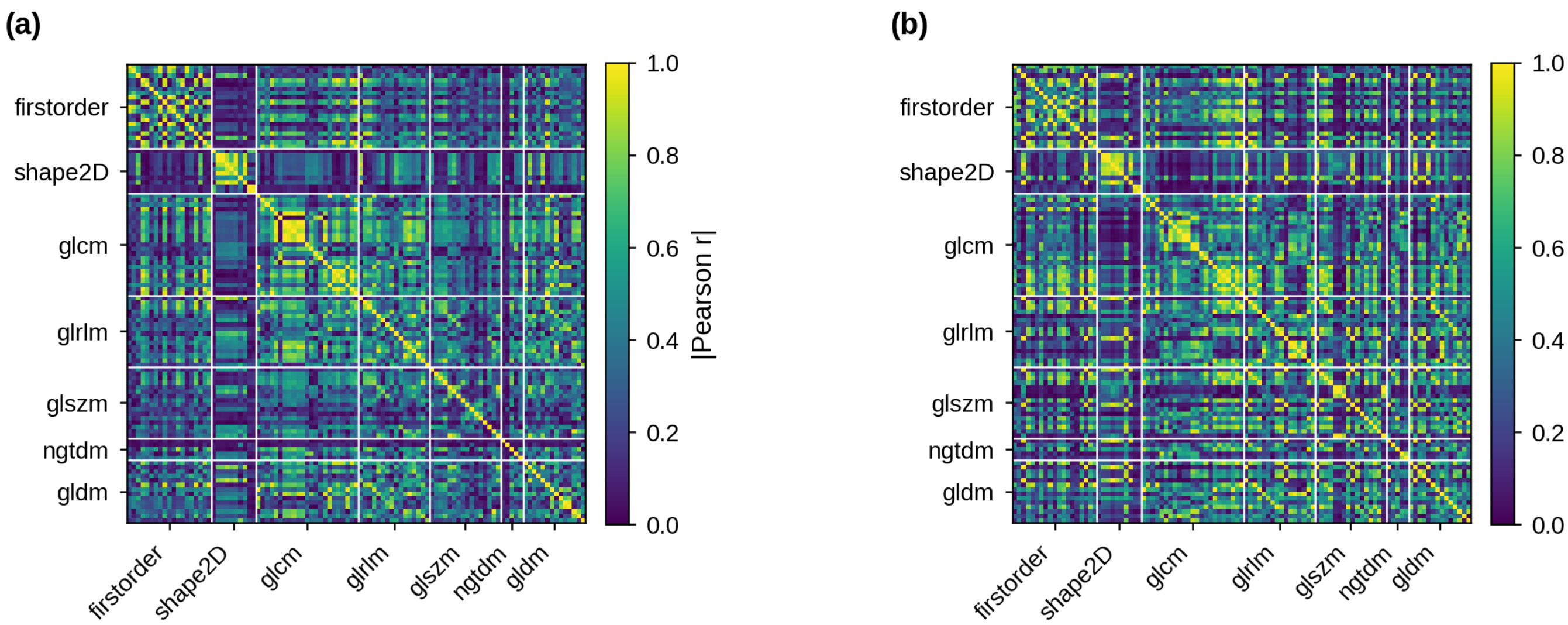


Figure S4: Absolute Pearson correlation between the 103 default QuRad features, ordered by class, on (a) the PUMA nuclei and (b) the TIGER regions. The matrices are computed on all objects for illustration, whereas the classifiers prune correlated features inside each training fold.

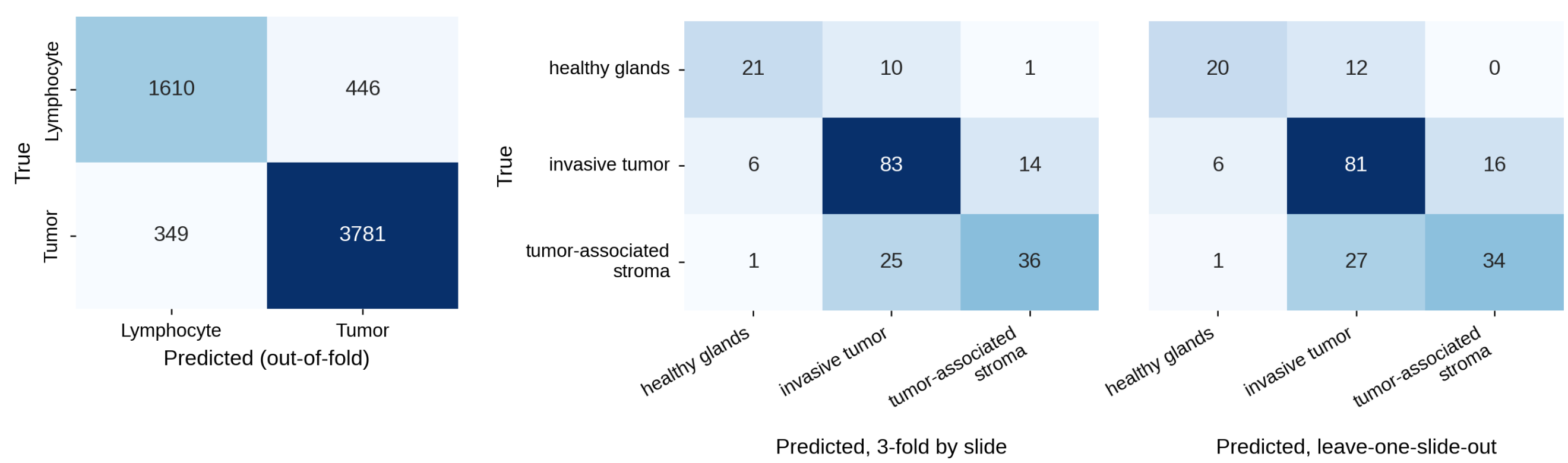


Figure S5: Out-of-fold confusion matrices. Left: PUMA, five folds grouped by tile. Middle and right: TIGER, three folds grouped by slide and leave-one-slide-out evaluation. Counts and per-object predictions are in the repository.
